# Designing and Testing Apps to Support Patients With Cancer: Looking to Behavioral Science to Lead the Way

**DOI:** 10.2196/12317

**Published:** 2019-04-22

**Authors:** Lauren M Hamel, Hayley S Thompson, Terrance L Albrecht, Felicity WK Harper

**Affiliations:** 1 Population Studies and Disparities Research Program Department of Oncology Wayne State University Detroit, MI United States

**Keywords:** behavioral science, cancer, mobile apps, evidence-based practice, smartphone, mHealth

## Abstract

**Background:**

Behavioral science has a long and strong tradition of rigorous experimental and applied methodologies, which have produced several influential and far-reaching theoretical frameworks and have guided countless inquiries of human behavior in various contexts. In cancer care, behavioral scientists have established a firm foundation of research focused on understanding the experience of cancer and using that understanding to design and implement theory- and evidenced-based interventions to help patients cope with the cancer experience. Given the rich behavioral research base in oncology, behavioral scientists are ideally positioned to lead the integration of evidence-based science on behavior and behavior change into the development of smartphone apps supporting patients with cancer. Smartphone apps are being disseminated to patients with cancer with claims of being able to help them negotiate areas of vulnerability in their cancer experience. However, the vast majority of these apps are developed without the rigor and expertise of behavioral scientists.

**Objective:**

In this article, we have illustrated the importance of behavioral science leading the development and evaluation of apps to support patients with cancer by providing an illustrative scientific process that our team of behavioral scientists, patient stakeholders, medical oncologists, and software developers used to empirically design and evaluate 2 patient-focused apps: the Discussion of Cost App (DISCO App) and MyPatientPal.

**Methods:**

Using a focused literature review and a descriptive roadmap of our team’s process for designing and evaluating patient-focused behavioral apps for patients with cancer, we have demonstrated how behavioral scientists are integral to the development of empirically sound apps to help support patients with cancer. Specifically, we have illustrated the process by which our multidisciplinary team combined the established user-centered design principles and behavioral science theory and scientific rigor to design and evaluate 2 patient-focused apps.

**Results:**

On the basis of initial acceptability and feasibility testing among patients and providers, our team has demonstrated how critical behavioral science is for designing and evaluating app-based interventions for patients with cancer.

**Conclusions:**

Behavioral science can and should be coupled with user-centered design principles to provide theoretical guidance and the rigor of the scientific method, thereby adding the much-needed and critical evidence for these types of app-based interventions for patients with cancer.

## Introduction

### Background

Behavioral scientists seek to understand the interpersonal and contextual motivations, and the limitations and parameters of those motivations, of human behavior. Guided by evidence-based theories and models, behavioral scientists apply the rigor of the scientific method to important areas of human behavior inquiry. For example, behavioral scientists have articulated, tested, and adapted several major theoretical frameworks (eg, Social Cognitive Theory [1], Theory of Planned Behavior [2], the Health Belief Model [3], and the Transtheoretical Model [4]) that have guided our systematic understanding of human behavior in myriad contexts, specifically in health contexts.

In cancer care, behavioral scientists have established a firm foundation of research focused on understanding the experience of cancer and using that understanding to design and implement theory- and evidence-based interventions for patients [5-9]. By integrating behavioral theories with the realities of patients’ cancer experience, behavioral scientists have helped improve care and outcomes for numerous patients with cancer, including those most likely to experience disparities in the quality of their care and their health outcomes [10].

### The Patient Experience

The National Comprehensive Cancer Network Distress Management Guidelines have identified areas of vulnerability that patients are likely to experience, including but not limited to the diagnostic workup, treatment planning, treatment for advanced disease, and when there is recurrence or disease progression [11,12]. Research by behavioral scientists has found that, for many patients, simply interacting with the health care system is a novel experience and may pose significant emotional and practical challenges for them and their families. For example, patients may struggle with effectively communicating with physicians and other providers [13-16], sharing in the decision making of their care plan [17,18], understanding complicated treatment regimens [19,20], and managing intrusive treatment side effects [21]. They may also have difficulty navigating a maze of appointments [22,23], frequently characterized by long wait times [24] and confusing physical surroundings, not to mention worrying about the financial burden of care [25]. Difficulties negotiating these areas of vulnerabilities can lead to poor patient satisfaction with care [15,17,18], increased psychological distress [12,21], missed or delayed appointments, and nonadherence to treatment [15].

### Leveraging Technology to Support Patients

Advances in smartphone apps have provided patients with readily available and low-cost tools to help manage their health [26,27] and, more importantly, navigate these areas of vulnerability [28-30]. In fact, apps are already being used to provide patients with information and strategies for prevention, detection, and management of treatments and side effects of cancer [31]. A recent review of apps specifically for patients diagnosed with breast cancer identified 599 unique apps. Unfortunately, less than 20% (118/599) of those apps included references to empirical studies or background source materials, highlighting the lack of an evidence base behind most patient-focused apps [32]. Another review of apps focusing on breast diseases, including breast cancer, evaluated whether apps were evidence-based, whether they had the involvement of a medical professional in the development, or had evaluated potential safety concerns. Of the 185 apps reviewed, only 11.4% (21/185) were evidence-based and only 10.3% (19/185) had the involvement of a medical professional in their development. Furthermore, 15.7% (29/185) of the apps had the potential to cause indirect harm to the consumer as they provided advice without documented evidence or medical professional input. Thus, although there is an abundance of apps available to support cancer patients, there is a remarkable lack of evidence underlying the content, underscoring the need for more scientific rigor in the development of apps for patients with cancer [33].

### Review of the Evidence

Recent published reviews of the effectiveness of app-based behavior change interventions are useful in identifying what aspects of intervention content are most important for behavior change. One such review conducted by Zhao et al evaluated 23 papers reporting on the effectiveness tests of mobile phone apps designed to improve various health issues [34]. They noted that only 6 of the 23 reviewed apps included a theory or model of behavior change (eg, Theory of Planned Behavior and Social Cognitive Theory). Apps that were designed with the guidance of a theory of behavior change were, however, more effective at influencing outcomes than those that lacked a theoretical basis. Another review that assessed behavior change communication interventions was silent on whether any theories or models of behavior change were included with any of the reviewed apps [35]. In sum, evidence is indicating that apps designed with the benefit of a behavioral theory or model as a guide will be more effective at prompting behavior change.

### The Role of Behavioral Science

As the findings from the review by Zhao et al demonstrate, behavioral scientists can play a unique and vital role in the design of app-based interventions and the evaluation of their effectiveness on key patient outcomes. An important exemplar is a report by Giunti et al on the design of an app to support patients with multiple sclerosis [36]. They described a user-centered design process guided by behavior change theories including the health belief model, goal setting theory, and self-determination theory. Similarly, Dicianno et al presented a roadmap of the design of a mobile health tool to promote goal achievement and self-management for patients with spina bifida and spinal cord injuries [37]. The intervention content, which focused on behavior change strategies, was informed by the self-determination theory, a theory of motivation to prompt behavior change. Although the effectiveness of these apps is yet to be published, they provided a promising genesis of the integration of behavioral science in app development.

Given the firm foundation of behavioral research in oncology, behavioral scientists are ideally positioned to lead the integration of evidence-based science on behavior and behavior change into the development of apps supporting patients with cancer. In this article, we have illustrated the contribution of behavioral science by describing the scientific process that our team of behavioral scientists, patient stakeholders, medical oncologists, and software developers used to design and test 2 patient-focused apps. This team collectively and empirically designed, built, and conducted acceptability testing of 2 apps designed to help patients with cancer at certain points in the cancer care continuum.

## Methods and Results

### Examples of Behavioral Science–Driven App Development and Evaluation

The *Discussion of Cost App* (*DISCO App,* built by CrossComm) [38] is a patient-focused app designed to help patients proactively manage their treatment costs through enhanced efficacy by (1) educating patients with cancer about the different types of treatment costs (eg, copayments, transportation, and lodging) and (2) prompting patient-oncologist treatment cost discussions. The premise of the app is that educating patients about treatment cost and prompting treatment cost discussions earlier in the treatment will help evoke proactive responses to financial needs and help prevent financial toxicity [39-41]. The DISCO App design was based on similar paper-based interventions (question prompt lists [QPLs]), which have been shown to effectively prompt active participation of the patient in treatment discussions [5,42] and facilitate patient-centered communication [13,42]. The design of QPLs is rooted in communication and social psychological theories of behavior of change, and their effectiveness has been tested in randomized controlled trials in several care settings [5,42].

The DISCO App advances traditional QPLs in 3 ways. First, it provides patients with a short educational video about treatment costs, about ways to manage those costs, and showing that discussing costs with their oncologist is an important first step. Second, it focuses on specific cost concerns (an emerging problem for many patients with cancer) previously shown to be important to patients with cancer [41]. Third, it uses patient-reported demographic information to provide a tailored list of cost-related questions. Before meeting their oncologist, patients are shown a short educational video about treatment costs in the DISCO App using an iPad and then the app has patients complete a short financial and demographic survey (eg, What is your annual household income?; How much does your health insurance cover?). On the basis of the patient’s responses, an individually-tailored list of cost-related questions is created. For example, patients may be prompted with the question “Is there someone I can talk to about my insurance and treatment cost questions?” Patients can then use these question prompts with their oncologist or other providers when discussing treatment cost.

*MyPatientPal* (built by CrossComm) is a patient app that is designed to help patients track and manage treatment side effects and medication adherence on a daily basis. The side effects of treatment (eg, pain, fatigue, diarrhea, and nausea) can be physically and emotionally debilitating and, when uncontrolled, can cause treatment complications, resulting in unscheduled care costs, patient’s out-of-pocket costs, and delays or discontinuation of treatment. Research shows that patient-reported outcomes (PROs), and in particular daily reporting of PROs, can help to identify significant changes in treatment-related side effects as well as quickly identify new and emerging side effects. On the basis of theories of self-management and self-efficacy [43-45], daily reporting of side effects and medications is theorized to increase patients’ self-efficacy for managing their own care, which in turn may increase adherence to medication and increase communication with providers about side effects. The app allows personalization of the daily diaries such that patients can select the specific medications and dosage and side effects (using items from the National Cancer Institute Common Terminology Criteria for Adverse Events [46]) that they and their providers want to track. The app also has a charting feature, which provides an easy-to-read display of patients’ daily reports, summarizing the intensity and frequency of side effects and medication use either by week or by month. Alongside allowing patients to see trends in their reports, these summaries can also be printed out and shared with providers to better inform their care.

In addition to being theoretically guided, both the DISCO App and MyPatientPal were developed using an iterative design-test-redesign process [47,48] in collaboration with a multidisciplinary team (eg, patients, providers, and software developers). First, the investigators discussed their app ideas with software experts in a university-based technology transfer department. Second, with guidance from the technology transfer department, the investigators conducted a series of customer discovery interviews (n=40 each) with key stakeholders (eg, on-treatment patients, survivors, caregivers, oncologists, and social workers) to determine (1) the relevance of the identified problem to stakeholders and (2) the extent to which the proposed app would help solve the problem. Responses from the discovery interviews were summarized and used to refine the content of the apps. Third, software designers in the technology transfer department developed wireframes. Wireframes provide a prototype of the structure and functions of a website or app and are often used at the initial phases of a build to allow redesign and refinement of the app. Wireframes also provide a roadmap for software developers by illustrating the various elements and screens of the app. Fourth, wireframes were reviewed with key stakeholders (eg, cancer survivors, clinicians, and social/behavioral scientists) for feedback on the design, content, and screen navigation. Finally, using that feedback, the investigators collaborated with a professional software development firm to revise the wireframes and use them to build the initial electronic prototype of the app, also known as a minimally viable product, to use in acceptability testing with key stakeholders. Using semistructured interviews, behavioral scientists then conducted qualitative interviews with stakeholders to review and provide feedback on the design and content of the app, its usefulness for patients, and suggestions for improvement. In the final stage, the investigators worked with the software development firm to create a final prototype (*build*) to use in feasibility and effectiveness testing in a clinic setting.

## Discussion

In summary, smartphone apps are being disseminated to patients with cancer with claims of being able to help them negotiate areas of vulnerability in their cancer experience. However, the vast majority of these apps are developed without the rigor and expertise of behavioral scientists.

### Principal Findings

To be sure, many apps benefit from user-centered design principles, which attend to how users interact with products and ensure they meet user needs. In contrast, behavioral scientists bring an important understanding of the psychological processes underlying the content and how the product can be used to effect behavior change, whether it is focused in health behaviors such as diet and exercise or self-management of diseases such as cancer. Thus, behavioral science has the potential to complement and even significantly augment user-centered design principles by providing theoretical guidance and the rigor of the scientific method, thereby adding the currently lacking but much needed empirical support for these types of apps.

### Conclusions

Thus, we argue that future apps designed to help patients with cancer should be built by a multidisciplinary team of experts including physicians, survivors, software developers, university technology transfer units, and behavioral scientists, who bring critical theoretical and evidence-based knowledge. This multidisciplinary approach means that app-based interventions will be user-friendly, evidence-based, and theoretically sound, and as such, more likely to be effective sources of support for patients with cancer through the myriad of issues and obstacles they will likely face. Furthermore, this type of team approach can lead to the development of patient-centered apps that meet the needs of stakeholders and improve the experience of cancer diagnosis, treatment, and survivorship for a diverse population of patients.

## Abbreviations

DISCO: Discussion of Cost
PRO: patient-reported outcome
QPL: question prompt list
